# Relationships Among Temporal Fine Structure Sensitivity, Transient Storage Capacity, and Ultra-High Frequency Hearing Thresholds in Tinnitus Patients and Normal Adults of Different Ages

**DOI:** 10.3389/fnagi.2022.869708

**Published:** 2022-04-26

**Authors:** Yu Ding, Yibo Liang, Chunmei Cao, Yueqi Zhang, Ming Hu

**Affiliations:** ^1^Division of Sports Science and Physical Education, Tsinghua University, Beijing, China; ^2^Department of Otorhinolaryngology Head and Neck Surgery, Tianjin First Central Hospital, Tianjin, China; ^3^Institute of Otolaryngology of Tianjin, Tianjin, China; ^4^Key Laboratory of Auditory Speech and Balance Medicine, Tianjin, China; ^5^Key Clinical Discipline of Tianjin (Otolaryngology), Tianjin, China; ^6^Otolaryngology Clinical Quality Control Centre, Tianjin, China

**Keywords:** tinnitus, temporal fine structure, break in interaural correlation, ultra-high frequency, aging

## Abstract

**Background:**

Elderlies and tinnitus patients often find it challenging to process acoustic signals in noisy environments. The sensitivity to temporal fine structure (TFS), the transient storage capacity for TFS, and the ultra-high frequency (UHF) thresholds are all associated with aging-related damage, evidenced by speech-in-noise perception deficits. In the present study, we aimed to investigate the relationships among TFS sensitivity, transient storage capacity, and UHF thresholds in tinnitus patients and normal adults of different ages.

**Methods:**

In the present study, 38 tinnitus patients (age ranging from 21 to 65) and 23 non-tinnitus adults (age ranging from 22 to 56) were enrolled, and some of their auditory indicators were examined, including the TFS-adaptive frequency (TFS-AF), break in interaural correlation (BIAC) delay threshold, and UHF thresholds.

**Results:**

We found no significant difference in TFS-AF thresholds and BIAC delay thresholds between the tinnitus group and normal group, while their relationships with age were more evident in the tinnitus group. Moreover, these two tests were only significantly correlated in the tinnitus group. UHF thresholds were significantly correlated with TFS-AF thresholds only in the tinnitus group, suggesting that the UHF hearing was positively associated with the TFS sensitivity.

**Conclusion:**

These findings indicated that the influencing factors, such as tinnitus and UHF thresholds, should be fully considered when examining age-related hearing decline, because the combination of tinnitus and poor UHF hearing might play a role in affecting hearing ability, such as TFS sensitivity.

## Introduction

Tinnitus refers to the feeling of conscious ringing in the ear without a corresponding external sound source or electrical stimulation. It is a common clinical symptom with increasing prevalence. A meta-analysis shows that the prevalence of tinnitus ranges from 11.9 to 30.3% using the same definition of tinnitus (McCormack et al., 2016). In this meta-analysis, 26 studies report the prevalence of tinnitus by different age groups and generally show an increasing prevalence with age. Therefore, tinnitus remains an essential underlying factor in determining the effect of aging on hearing ability.

Many people with normal audiograms have hearing problems, and tinnitus patients show impaired speech-in-noise (SiN) perception (Gilles et al., 2016). Deficits in speech perception for tinnitus patients and/or older people with normal audiograms probably reflect that age and tinnitus are independent factors associated with poorer speech perception. Animal studies indicate that the “hidden hearing loss” phenomenon may be related to the massive loss of inner hair cell synapses caused by acoustic overexposures (Liberman and Liberman, 2015). Additionally, hearing loss can occur in higher frequency ranges, and studies have found that a majority of tinnitus patients with normal hearing thresholds up to 8 kHz have hearing impairment above 8 kHz (Vielsmeier et al., 2015), which is also common in the non-tinnitus population (Waechter et al., 2021). The ability to process temporal fine structure (TFS) information, including the sensitivity (Moore and Sek, 2009; Moore et al., 2012; Perez Vallejos et al., 2014; Füllgrabe and Moore, 2018) and storage capacity (Huang et al., 2009; Li et al., 2013; Liu et al., 2016) of TFS, is another aspect of suprathreshold processing that has received considerable attention in recent years.

In the cochlea, broadband sound, such as speech, is decomposed into several narrowband signals, and these signals can be considered as the relatively slow variation in amplitude over time (envelope, ENV) and the rapid oscillations with a rate close to the center frequency of the band (TFS) (Moore and Sek, 2009). The processing ability of TFS is essential for basic auditory functions, such as unmasking and localization of sound source. ENV is most important for speech perception, and TFS is most important for sound localization and pitch perception, revealing the acoustic basis for the “what” and “where” pathways in the auditory cortex (Smith et al., 2002).

Among the many auditory-related indicators, the ability related to TFS declines quickly with age. By using the TFS1 test (Moore et al., 2012), TFS2 test (a modified version of the TFS1 test by using a narrower passband) (Hopkins and Moore, 2011), TFS-LF (low-frequency) test (Füllgrabe and Moore, 2018), and TFS-AF (TFS-adaptive frequency) test (Füllgrabe et al., 2018), studies have shown that TFS sensitivity declines with age in the absence of elevated audiometric thresholds or broadened auditory filters. Among these methods, the TFS-AF test has many advantages and is the most recommended measurement of fine structure sensitivity (Füllgrabe et al., 2018). Multiple factors can predict TFS sensitivity. A meta-analysis shows that audiometric threshold and age only account for up to 42% of the variance in binaural TFS sensitivity, leaving a substantial variance to be explained by other factors, such as cognitive abilities (Füllgrabe and Moore, 2018). Among various cognitive abilities, working memory capacity is involved in temporal auditory processing (Troche and Rammsayer, 2009; Broadway and Engle, 2011). Studies have found that working memory is related to the high-frequency hearing threshold (10–16 kHz), while the presence of tinnitus may not impair the working memory test (Waechter et al., 2019, 2021).

Similarly, the storage capacity for TFS (as primitive auditory memory in many studies, PAM) also declines with age (Li et al., 2009). In a noisy and reverberant environment, people can still recognize and understand the voice of the target speaker. This common phenomenon reflects the remarkable ability of the brain to use various spatial (and/or non-spatial) cues to facilitate selective attention to target speech and follow the target stream against irrelevant influences (Cherry and Colin, 1954). To perceptually separate the target signal from other disruptive sound signals, the auditory system needs to separate the sound of the target and interfering sources and integrate the target sound wave that directly comes from the target source with its reflections, depending on the similarity between the leading direct and the lagging reflections (Li et al., 2005; Huang et al., 2008). However, due to the direct-reflection delay, faithful storage of TFS signals of the leading sound waves in the central auditory system is required for the computation of the similarity (Huang et al., 2009). Theoretically, faithful storage of TFS signals of the leading wave is necessary for both the central computation of the similarity and the perceptual integration between the leading and lagging waves. This faithful auditory storage of TFS has been recognized as the early point in the chain of the transient auditory memory system and termed as PAM (Li et al., 2013).

Primitive auditory memory can be measured using behavioral methods. Humans are extremely sensitive to the dynamic changes in interaural correlation, such as detecting a dynamic break in interaural correlation (BIAC, a brief drop of interaural correlation from 1 to 0 and then return to 1) in a steady-state noise (Akeroyd and Summerfield, 1999; Boehnke et al., 2002). Introducing a change in interaural correlation does not alter the monaural energy spectrum of the sound signals but changes dichotic repetition pitch (Bilsen and Goldstein, 1974) and the loudness (Moore, 2003) of the noise. Furthermore, even if a binaural time interval is introduced, humans can still detect the presence of BIAC (Li et al., 2013). By increasing the time interval, a threshold can be found, and participants cannot detect BIAC beyond this threshold. This measured binaural delay threshold represents the ability of PAM, which is the maximum retention time of TFS (Kong et al., 2012, 2015).

Taken together, in the normal population, both sensitivity and storage capacity for TFS decline with age, and these studies generally do not consider the effects of tinnitus or ultra-high frequency (UHF) threshold. In the present study, we examined the relationships among age, the sensitivity and storage capacity of TFS, and the UHF threshold in the tinnitus group and normal group.

## Materials and Methods

### Participants

A total of 61 adults participated in this study, including 38 patients with chronic subjective tinnitus (19 males and 19 females, age ranging from 21 to 65, with a mean of 45.08 years and a standard deviation of 11.04) examined in Tianjin First Central Hospital (Tianjin, China), and 23 non-tinnitus people (11 males and 12 females, age ranging from 22 to 56, with a mean of 35.26 years and a standard deviation of 11.26) from nearby communities. Kolmogorov-Smirnov tests showed that age obeyed a normal distribution in both tinnitus group and normal group (for tinnitus group: K-S Z scene = 0.771, *p* = 0.591; for normal group: K-S Z score = 0.766, *p* = 0.600).

Each participant underwent otoscopy and immittance measures by a certified audiologist in otoscopy and tympanometry. Otoscopic examinations had to reveal clear ear canals for all participants. Tympanograms were carried out using a 0.226-kHz probe tone and a pressure change in the ear canal equal to 200 daPa/sec. Resultant peak admittance, peak pressure, tympanometric width, and ear canal volumes had to be within normal ranges. Participants had to have a detectable acoustic reflex (>0.02 mmho) at 1 kHz evoked by contralateral stimulation. Pure-tone thresholds were collected by certified audiologists using pulsed tones at 125 Hz, 250 Hz, 500 Hz, 1, 2, 4, and 8 kHz. UHF behavioral thresholds were obtained at 10, 12.5, 16, and 20 kHz.

Written informed consent was obtained from all participants, and a modest stipend was given for their participation. The study was approved by the Tsinghua University Ethics Committee.

### Apparatus and Stimuli

Each participant sat comfortably in a chair in a sound-attenuated room, and the test environment was in compliance with the requirements of ISO 8253-1:2010. All the acoustic signals, calibrated by a sound-level meter (AUDit and System 824, Larson, Davis, CA, United States), were delivered using the Creative Sound Blaster (Sound Blaster X-Fi Surround 5.1 Pro, Creative Technology Ltd., Singapore) and presented to participants over the two earpieces of Sennheiser HD650 headphones.

Daily calibration was completed for each pair of earphones (conventional and UHF transducers) using electro-acoustic ear simulators. A complete acoustic calibration (industry standard) of all equipment was performed prior to, twice during, and after data collection. Tympanometry was completed using GSI TympStar Pro. Conventional behavioral thresholds and high-frequency behavioral thresholds were obtained with GSI AudioStar Pro audiometers.

All participants were subjected to test for pure-tone hearing threshold first. The order of the BIAC delay threshold and TFS-AF tests was randomized between participants. Before each test, there would be a practice phase to ensure that participants understood the experimental task (details of the practice phase are described below).

### Test for Break in Interaural Correlation Delay Threshold

The storage capacity of TFS was determined by the BIAC delay threshold test. The parameters and procedures of the BIAC delay threshold test have been described in detail in previous studies (Li et al., 2013; Lei and Ding, 2021). In the testing stage of the BIAC delay threshold, Gaussian wideband noises (2,000 ms in duration, including 30-ms rise-fall time) were synthesized using the “randn()” function in the MATLAB function library (the Math Works Inc., Natick, MA, United States) at the sampling rate of 48 kHz with a 16-bit resolution. The intensity of the noise stimulus was set at 60 dB sound pressure level (SPL). In one presentation, the left-headphone noise was an exact copy of the right-headphone noise. In the other presentation, the left-headphone noise was also identical to the right-headphone noise except that its temporal middle was substituted with a randomly selected independent noise fragment (e.g., the BIAC) with a fixed duration of 200 ms before filtering. Therefore, a brief break of interaural correlation, from 1 to 0 and then return to 1, was introduced. The offset-to-onset interval between the two presentations was 500 ms. In each presentation, the noise presented through the right headphone always started simultaneously with or led that presented through the left headphone.

Before the BIAC test, all the participants became familiarized with binaurally presented noise either with or without the BIAC. Subsequently, the participant initiated a trial by pressing the computer mouse, and the task was to identify which of the two presentations contained the BIAC. The longest interaural interval (IAI) for BIAC detection was measured using a three-up-one-down paradigm (Levitt, 1971). The IAI started from 0 ms, and then it was increased following three consecutive correct identifications of the presentation containing the BIAC and decreased following one incorrect identification. The initial step size of changing the IAI was 16 ms, which was altered by a factor of 0.5 with each reversal of direction until the minimum size of 1 ms was reached. Visual feedback was given after each trial to indicate whether the identification was correct or not. The test session was terminated following 10 reversals in direction, and the longest IAI was defined as the mean IAI for the last six reversals.

### Test for the Sensitivity of Temporal Fine Structure

In the testing stages of TFS-AF, this study used a special software package published by Moore on the Internet^1^ (Sęk and Moore, 2020) with most parameters at the default settings (Sęk and Moore, 2012).

Temporal fine structure-adaptive frequency test is a new method to determine the binaural sensitivity to TFS (Füllgrabe et al., 2017). Two consecutive intervals were presented on each trial, separated by 500 ms. Each interval contained four consecutive 400-ms tones, separated by 100 ms. In one interval, the first and third tones were the same, while the second and fourth tones differed in their interaural phase difference (IPD) by φ (the target). Participants sensitive to binaural TFS perceive pure tones with a sufficiently large IPD lateralized toward one ear. In the other interval, the IPD of all tones was always 0° (the standard), while tones with IPD = 0° were perceived as emanating from close to the center of the head. Participants were asked to indicate which of the two intervals contained a sequence of tones that appeared to move within the head. The frequency was adaptively adjusted. The TFS-AF test used a two-up-one-down to estimate the 71% correct point on the psychometric function. If the participants chose twice in the right choices, the frequency would increase, and otherwise, it would decrease. The initial magnification was changed by 1.4 times, the first reversal was 1.2 times, and the third and subsequent times were 1.1 times. The test was terminated after eight reversals, and the geometric average of the last six inflection points was used as the threshold estimate. For the TFS-AF test in this study, the initial frequency was 200 Hz, the sound intensity of the left and right ears was 30 dB SPL, and the phase difference (φ) was set to 180°.

In the practice run of the TFS-AF test, the participants could choose to listen to four tone bursts (200 Hz) all with an IPD of 0° (“Not Moving”) or to four bursts that alternated between an IPD of 0° and an IPD of 180° (“Moving”). When the participant was satisfied that they could hear the difference between “Not Moving” and “Moving,” they could finish the practice run. All the results were automatically output by the software after the test.

It should be noted that the BIAC delay threshold test used the three-up-one-down procedure. In contrast, the TFS-AF test used the two-up-one-down procedure, which was consistent with past studies and could facilitate horizontal comparison with the results of past studies.

## Results

### Ultra-High Frequency Hearing Thresholds in Healthy Controls and Tinnitus Patients

**FIGURE 1:**
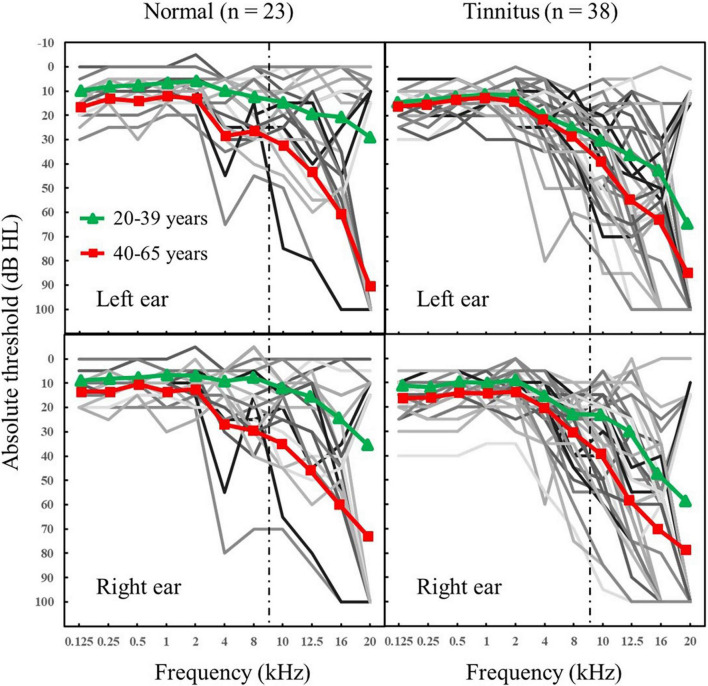
Individual (thin lines) and mean (filled symbols and thick lines) audiometric thresholds (in dB hearing level [HL]) for frequencies between 0.125 and 20 kHz for each ear and two groups: normal group without any tinnitus; tinnitus group. To the right of the dashed line is the UHF range. Triangle symbol: 20–39 years; Square symbol: 40–65 years.

Air-conduction pure-tone audiometric thresholds were assessed following the procedure recommended by the British Society of Audiology (2004) and using standard calibrated audiometric equipment. Figure 1 presents the results of pure-tone hearing thresholds for each participant. We included the UHF range in the measurement of pure-tone hearing. Several participants with poor pure-tone hearing were not excluded, in order to present the data as completely as possible. After excluding four participants whose pure-tone hearing threshold below 4 kHz was higher than 35 dB, the experimental results of this study remained unchanged.

**FIGURE 2:**
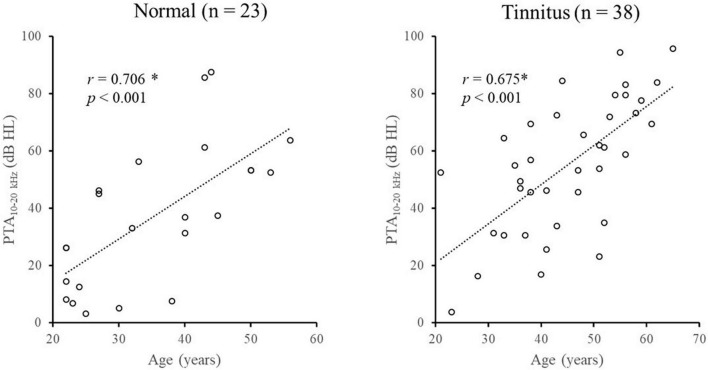
Illustration of the age-related changes in PTA_10–20 kHz_ in the normal group and tinnitus group. *Significant effect after Bonferroni’s correction, *p* < 0.008.

Spearman correlation analyses showed that the pure-tone averages (PTA) were significantly decreased with age in both the normal group (for PTA_0_._125–1 *kHz*_: *r* = 0.597, *p* = 0.003; for PTA_2–8 *kHz*_: *r* = 0.596, *p* = 0.003; for PTA_10–20 *kHz*_: *r* = 0.706, *p* < 0.001) and tinnitus group (for PTA_0_._125–1 *kHz*_: *r* = 0.362, *p* = 0.025; for PTA_2–8 *kHz*_: *r* = 0.384, *p* = 0.017; for PTA_10–20 *kHz*_: *r* = 0.675, *p* < 0.001). Figure 2 shows the age-related changes in PTA_10–20 *kHz*_ in the normal group and tinnitus group. Overall, the correlations between age and pure-tone averages were systematically lower in the tinnitus group than in the normal group.

### Distribution of Temporal Fine Structure-Adaptive Frequency and Break in Interaural Correlation Delay Thresholds With Age

**FIGURE 3:**
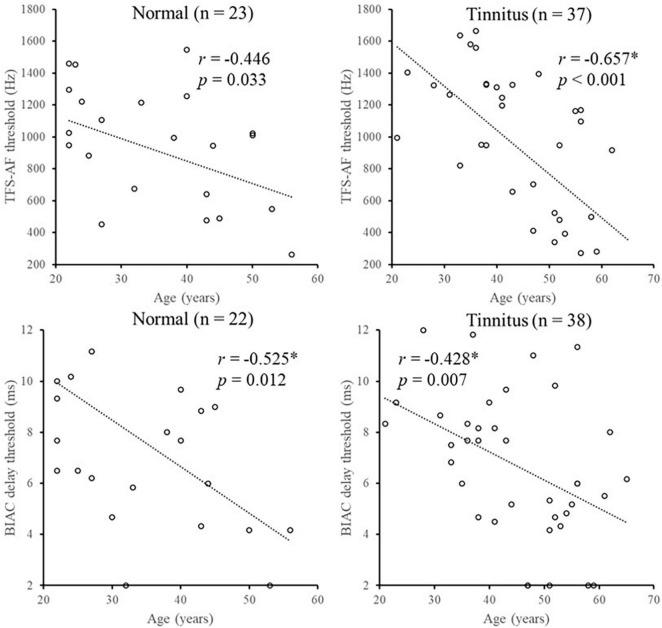
Illustration of the correlation analysis of the BIAC delay threshold and TFS-AF test with age. *Significant effect after Bonferroni’s correction, *p* < 0.013.

One participant in the normal group failed to complete the BIAC delay threshold test, and one participant in the tinnitus group failed to complete the TFS-AF test. These two participants were excluded from subsequent analyses involving correlation. Kolmogorov-Smirnov tests showed that both the BIAC delay threshold and TFS-AF threshold followed a normal distribution (for BIAC: K-S Z scene = 0.713, *p* = 0.690; for TFS-AF: K-S Z score = 0.881, *p* = 0.419). Two independent sample *t*-tests did not find any significant difference in both the BIAC delay thresholds and TFS-AF thresholds between the tinnitus group and normal group (for BIAC: *t*(58) = 1.026, *p* = 0.309; for TFS-AF: *t*(58) = 0.062, *p* = 0.950). For the normal group, the mean BIAC delay threshold was 7.64 ms (SD = 4.50 ms), and the mean TFS-AF threshold was 914.83 Hz (SD = 393.77 Hz). For the tinnitus group, the mean BIAC delay threshold was 6.67 ms (SD = 2.81 ms), and the mean TFS-AF threshold was 907.28 Hz (SD = 488.66 Hz). Figure 3 shows the distribution of the BIAC delay threshold and TFS-AF threshold at different ages. Four independent Spearman correlation analyses were used. After Bonferroni’s correction, the BIAC delay threshold was significantly decreased with age in the normal group. Both the BIAC delay threshold and TFS-AF threshold were significantly decreased with age in the tinnitus group.

### The Relationship Between Temporal Fine Structure-Adaptive Frequency and Break in Interaural Correlation Delay Thresholds

**FIGURE 4:**
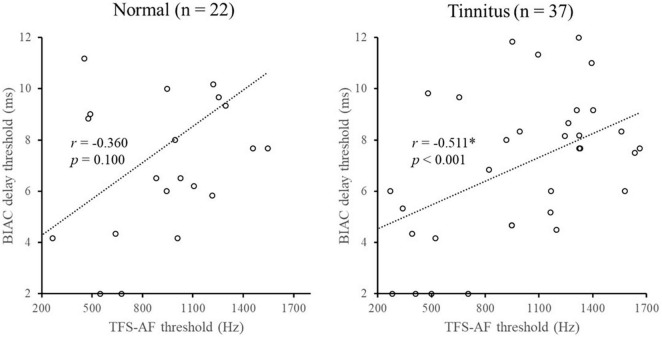
Illustration of the correlation analysis of the BIAC delay threshold and TFS-AF test. *Significant effect after Bonferroni’s correction, *p* < 0.025.

Temporal fine structure-adaptive frequency measures the sensitivity of TFS, while the BIAC delay threshold test mainly determines the preservation capacity of TFS. Therefore, measured scores of BIAC delay thresholds may be related to TFS sensitivity. To explore such a possibility, we analyzed the relationship between the BIAC delay threshold and TFS-AF threshold using Spearman correlations. Figure 4 shows that the TFS-AF threshold was significantly correlated with the BIAC delay threshold in the tinnitus group but not the normal group. This finding might be attributed to the fact that some patients in the tinnitus group had a significant decrease in TFS sensitivity and storage capacity, leading to a higher correlation between the BIAC delay thresholds and TFS-AF thresholds.

### The Relationship Between Ultra-High Frequency Hearing Thresholds and Temporal Fine Structure-Adaptive Frequency and Break in Interaural Correlation Delay Threshold

Studies have explored the relationship between TFS-AF and pure-tone hearing below 8 kHz (Füllgrabe et al., 2018). However, pure-tone hearing above 8 kHz may also affect the processing of TFS. Correlations were calculated between BIAC delay thresholds, TFS-AF thresholds, and UHF hearing thresholds for the normal group and tinnitus group to explore other potential contributors to the individual variability in BIAC delay thresholds and TFS-AF thresholds. Spearman correlations were used to avoid non-normal distribution. Table 1 shows the results and associated significance levels. Spearman correlation analysis showed that the TFS-AF threshold was significantly correlated with the PTA of UHF threshold in the tinnitus group, suggesting that UHF hearing was positively associated with TFS sensitivity.

**TABLE 1:** Spearman’s r (with associated two-tailed significance levels in parentheses) between individual TFS-AF thresholds, BIAC delay thresholds, and UHF hearing thresholds for the normal group (*n* = 37) and tinnitus group (*n* = 22).

		PTA_0.125–1 kHz_	PTA_2–8 kHz_	PTA_10–20 kHz_
*normal*	BIAC delay threshold (ms)	−0.237 (0.288)	−0.174 (0.440)	−0.403 (0.063)
	TFS-AF threshold (Hz)	−0.335 (0.118)	−0.048 (0.830)	−0.301 (0.163)
*tinnitus*	BIAC delay threshold (ms)	−0.455 *(0.004)	−0.094 (0.574)	−0.323 (0.048)
	TFS-AF threshold (Hz)	−0.381 (0.020)	−0.286 (0.087)	−0.476* (0.003)

PTA, pure-tone averages. *Significant effect after Bonferroni’s correction, p < 0.004.

## Discussion

In noisy, reverberant environments, the elderlies often find it challenging to process acoustic signals, even though they can function well in quiet environment (Stuart and Phillips, 1996). Many studies have analyzed possible causes and influencing factors from different perspectives. First, the sensitivity to monaural/binaural TFS is poorer for elderlies compared with younger adults, and this may contribute to age-related declines in the ability to understand speech in noisy situations (Hopkins and Moore, 2011; Moore et al., 2012; Füllgrabe et al., 2015; Füllgrabe and Moore, 2018). Second, detecting a change in correlation is an essential component of scene parsing. For correlation comparison, temporary storage of sound’s waveform (mainly TFS) is necessary (Li et al., 2013). Third, the representation of aspects of the TFS decays more rapidly in elderlies compared with younger adults (Li et al., 2009), suggesting that elderlies can not be as capable as younger adults in parsing auditory scenes. Finally, tinnitus patients show decreased SiN reception (Gilles et al., 2016), and high-frequency audiometry (>8 kHz) provides relevant additional information in tinnitus patients using conventional audiometry (Vielsmeier et al., 2015). Exploring the relationship between these factors can help understand age-related auditory perception changes in noisy situations.

Recently, more attention has been paid to the symptoms of tinnitus due to the increasing number of tinnitus patients (Dawes et al., 2014). Auditory acuity above 8 kHz is significantly associated with speech intelligibility in noise (Yeend et al., 2017; Zadeh et al., 2019; Trine and Monson, 2020), and UHF impairment may be associated with tinnitus (Vielsmeier et al., 2015). In addition, many hearing-related studies indicate that the participants have normal pure-tone hearing thresholds. Some of these participants may have hearing loss in the UHF range, affecting the measurement of some hearing indicators.

Several recent studies have reported that tinnitus patients and normal controls perform comparably well in cognitive tests when the hearing status is controlled (Waechter and Brännström, 2015; Waechter et al., 2019, 2021; Glick and Sharma, 2020; Hamza and Zeng, 2021; Jensen et al., 2021). In addition, part of the variance in TFS sensitivity is associated with factors such as cognitive abilities (Füllgrabe and Moore, 2018), suggesting that the tinnitus group and normal group do not differ significantly in terms of TFS sensitivity.

In the present study, the BIAC delay threshold was correlated with age in both the normal group and tinnitus group, which was consistent with previous studies (Li et al., 2009). Studies have found that younger listeners can detect significantly shorter BIACs compared with elderlies, and younger listeners can detect BIAC at significantly longer interaural delays (Li et al., 2009). When the noise arriving at one ear is delayed relative to that at the other ear, elderlies can not detect the BIAC as readily as younger adults, suggesting that elderlies have worse sound’s waveform (mainly TFS) storage capacity, which indicates that the representation of aspects of the sound’s waveform decays more rapidly in elderlies compared with younger listeners. Moreover, these age-related deficits are not associated with listeners’ audiograms in previous studies (Li et al., 2009). In the present study, there was no significant correlation between PTA and BIAC delay threshold in the normal group, which was consistent with the previous studies. However, there was a substantial correlation between PTA (0.125–1 kHz) and BIAC delay threshold in the tinnitus group, suggesting the importance of identifying subjects’ tinnitus in auditory research, especially in studies on the influence of aging.

Studies have investigated the sensitivity to binaural TFS changes across the older age range. The TFS-AF scores for elderlies are significantly poorer compared with young adults, which are decreased by about 162 Hz on average for each 10-year increase in age over the range from 60 to 85 years (Füllgrabe et al., 2018). In the present study, we found that TFS-AF scores were associated with the BIAC delay threshold in the tinnitus group and also associated with UHF hearing thresholds (10–20 kHz). However, the variability and relationship of these indicators are different in different populations. Further details of the participants can be identified as much as possible in future research.

Performance on binaural tests, such as the BIAC delay threshold test and TFS-AF test, may depend partly on the monaural coding of TFS information before binaural interaction (Füllgrabe et al., 2017; Whiteford et al., 2017). Loss of inner hair cells can lead to more “noisy” TFS, which may be an age-dependent change that affects the processing of monaural TFS information (before the point in the auditory pathway where binaural interaction occurs) (Makary et al., 2011; Sergeyenko et al., 2013; Füllgrabe et al., 2018). On average, each inner hair cell and 3 to 4 outer hair cells form an acoustic frequency unit. Some studies suggest that tinnitus can be attributed to damage to the outer hair cells of the cochlea (Sztuka et al., 2010), and the occurrence of tinnitus with normal hearing may be an early signal of hearing loss and early damage of cochlear hair cells. Collectively, damage to hair cells might be one of the possible explanations of some correlation results in this study.

Several recent studies have proposed theoretical models based on stochastic resonances, which argue that adding neuronal internal noise to the system can counteract hidden and/or non-hidden hearing loss, and the development of a tinnitus percept is a side effect of this process (Krauss et al., 2016, 2018; Krauss and Tziridis, 2021; Schilling et al., 2021). Stochastic resonance models can explain some of the results of this study. In this study, hearing threshold and age showed a lower correlation in the tinnitus group, which may be the benefit of internal noise (Gollnast et al., 2017). To a certain extent, the BIC delay threshold reflects the ability to process the correlation (Li et al., 2013), and in the stochastic resonance model, auto-correlation of the sensor output is crucial for finding the optimal noise level that maximizes the mutual information between sensor input and output (Krauss et al., 2017). Future studies can explore these models and mechanisms to explain potential relationships between these auditory indicators.

### Limitations

The pathogenesis of tinnitus is complex, many factors can affect the occurrence of tinnitus, and different types of tinnitus may have different damage mechanisms. Due to the insufficient number of participants, further distinguishing the types of tinnitus would lead to insufficient testing power. Therefore, we could not conduct a more detailed discussion of the mechanism. The number of participants should be increased in future studies to further differentiate the types of tinnitus.

Some older participants (especially in the tinnitus group) had very poor test scores. Although they were able to complete the practice sessions and the early stages of the tests, the final results were very poor. Therefore, the analysis of the correlation was inevitably affected by extreme values. Although we used non-parametric tests (Spearman correlation analysis) to reduce the influence of extreme values, there was still a large part of the variability that could not be well explained. Future research will further refine the influencing factors and expand the number of participants to reduce this variability. This study presented all data points in scatter plots for reference.

## Conclusion

In the present study, we examined the age-dependent changes of three types of auditory indicators, including TFS-AF thresholds, BIAC delay thresholds, and UHF hearing thresholds, in the normal group and tinnitus group. Among them, TFS-AF thresholds and BIAC delay thresholds had no significant difference between the two groups, while their relationship with age was more obvious in the tinnitus group. TFS-AF thresholds and BIAC delay thresholds reflect sensitivity and transient storage capacity of TFS, respectively, and the scores of these two tests were only significantly correlated in the tinnitus group. Correlations between UHF hearing thresholds and TFS-AF thresholds were observed only in the tinnitus group. Overall, the above results suggested that the influencing factors, such as tinnitus, should be fully considered when examining age-related hearing ability decline because the hearing ability of the tinnitus group might have greater variability and more obvious degradation.

## Data Availability Statement

The raw data supporting the conclusions of this article will be made available by the authors, without undue reservation.

## Ethics Statement

The studies involving human participants were reviewed and approved by the Tsinghua University Ethics Committee. The patients/participants provided their written informed consent to participate in this study.

## Author Contributions

YD and MH: conception, design, acquisition of data, analysis of data, interpretation of data, writing – original draft, writing, review, and editing. YL: conception, design, data acquisition, data analysis, data interpretation, writing, review, and editing. CC: conception, design, funding acquisition, writing, review, and editing. YZ: data acquisition, data analysis, data interpretation, writing, review, and editing. All authors contributed to the article and approved the submitted version.

## Conflict of Interest

The authors declare that the research was conducted in the absence of any commercial or financial relationships that could be construed as a potential conflict of interest.

## Publisher’s Note

All claims expressed in this article are solely those of the authors and do not necessarily represent those of their affiliated organizations, or those of the publisher, the editors and the reviewers. Any product that may be evaluated in this article, or claim that may be made by its manufacturer, is not guaranteed or endorsed by the publisher.

## Footnotes

1
https://www.psychol.cam.ac.uk/hearing
